# Association between *IGF2BP2* Polymorphisms and Type 2 Diabetes Mellitus: A Case–Control Study and Meta-Analysis

**DOI:** 10.3390/ijerph13060574

**Published:** 2016-06-09

**Authors:** Ping Rao, Hao Wang, Honghong Fang, Qing Gao, Jie Zhang, Manshu Song, Yong Zhou, Youxin Wang, Wei Wang

**Affiliations:** 1 School of Public Health, Capital Medical University, Beijing 100069, China; raopingbj@126.com (P.R.); wanghaostudy@163.com (H.W.); fanghonghong79@sina.com (H.F.); shouyigaoqing@163.com (Q.G.); zhangjie@ccmu.edu.cn (J.Z.); songms@ccmu.edu.cn (M.S.); 2 Beijing Institute of Heart, Lung and Blood Vessel Diseases, Beijing Anzhen Hospital, Capital Medical University, Beijing 100029, China; yongzhou78214@163.com; 3 School of Medical Sciences, Edith Cowan University, Perth, WA 6027, Australia

**Keywords:** type 2 diabetes mellitus, *IGF2BP2*, case-control study, meta-analysis, Asian

## Abstract

*Background:* Genome-wide association studies (GWAS) found that *IGF2BP2* rs4402960 and rs1470579 polymorphisms were associated with type 2 diabetes mellitus (T2DM) risk. Many studies have replicated this association, but yielded inconsistent results. *Materials and Methods:* A case-control study consisting of 461 T2DM patients and 434 health controls was conducted to detect the genetic susceptibility of *IGF2BP2* in a northern Han Chinese population. A meta-analysis was to evaluate the association more precisely in Asians. *Results:* In the case-control study, the carriers of TT genotype at rs4402960 had a higher T2DM risk than the G carriers (TG + GG) (adjusted odd ratio (AOR) = 1.962, 95% confidence interval (95% CI) = 1.065–3.612, *p* = 0.031]; CC carriers at rs1470579 were more susceptible to T2DM than A carriers (CA + AA) (AOR = 2.014, 95% CI = 1.114–3.642, *p* = 0.021). The meta-analysis containing 36 studies demonstrated that the two polymorphisms were associated with T2DM under the allele comparison, genetic models of dominant and recessive in Asians (*p* < 0.05). The rs4402960 polymorphisms were significantly associated with the T2DM risk after stratification by diagnostic criterion, size of sample and average age and BMI of cases, while there’re no consistent results for rs1470579. *Conclusions:* Our data suggests that *IGF2BP2* polymorphisms are associated with T2DM in Asian populations.

## 1. Introduction

The rapidly increasing prevalence of type 2 diabetes mellitus (T2DM) is identified as a major international health concern, with a great impact on global morbidity and premature mortality, not to mention the pressure on society and the economy of its chronic complications [1]. In China, the prevalence of diabetes increased from 0.9% in 1980 to 11.6% in 2013, suggesting that there were approximately 113.9 million Chinese adults suffering from diabetes [2].

T2DM is a complex metabolic syndrome with a strong genetic component, which plays a role in the initiation and development of T2DM [3,4]. Over the past several years, the genome-wide association studies (GWAS) have identified approximately 40 susceptibility loci [5]. Since the association of the gene insulin-like growth factor 2 mRNA binding protein 2 (*IGF2BP2*) and risk of T2DM was founded by the study group of the Wellcome Trust Case Control Consortium (WTCCC), *IGF2BP2* has been identified as a notable T2DM candidate gene [6,7,8].

*IGF2BP2* is located on chromosome 3q27 [7]. *IGF2BP2*, highly expressed in pancreatic islets, belongs to a family of the insulin-like growth factor 2 (*IGF2*) mRNA-binding proteins, which play roles in normal embryonic growth and development [9]. Moreover, *IGF2BP2* has been found to be associated with decreased insulin secretion, which plays a role in T2DM [10]. Duesing *et al.* stated that rs4402960 and rs1470579 of *IGF2BP2* gene associated with T2DM susceptibility by a comprehensive genetic association research [11]. However, Cui *et al.* reported no association of the single nucleotide polymorphisms (SNPs) with diabetes in a Han Chinese population [12]. Moreover, subsequent replication studies yielded conflicting or inconclusive results [13,14,15,16].

The risk allele frequencies of the T2DM susceptible locus are different among populations, for example distributions of T allele in rs4402960 are 35% in Asians and 30% in Caucasians. In addition, the insufficient power and small effect of the polymorphisms on T2DM risk may explain to some degree the inconclusive results. In an effort to arrive at a more definitive conclusion, we conducted a case-control study to analyze the associations between rs4402960 and rs1470579 of the *IGF2BP2* gene polymorphisms and T2DM in a group of northern Han Chinese subjects followed by a meta-analysis in the Asian population.

## 2. Experimental Section

### 2.1. Study Participants

The present study was conducted in accordance with the Declaration of Helsinki guidelines. Written informed consents were obtained from all the participants. The study was approved by the Ethics Committee of Capital Medical University (approval number 2013SY30). In this case-control study, 461 T2DM-diagnosed patients and 434 controls were recruited at the Jidong Oil Field Hospital (Hebei, China) from January 2009 to October 2013. The T2DM patients were diagnosed and confirmed by the American Diabetes Association criteria [17] or had a documented clinical diagnosis of T2DM from clinical records. The health controls who did not previously diagnosed diabetes, and had fasting glucose values <5.6 mmol/L without glucose-lowering medications were recruited from the same area and matched by gender with the cases. Potential participants who had impaired renal function, malignancies or connective tissue disease were excluded from this study.

### 2.2. SNPs Genotyping

Genomic DNA was extracted from peripheral white blood cells using blood genome DNA extraction kits according to the manufacturer’s instructions (BioTeke, Beijing, China). SNPs were genotyped by matrix assisted laser desorption/ionization-time-of-flight mass spectrometry (MALDI-TOF MS) using a Mass ARRAY system (Sequenom, Inc., San Diego, CA, USA). The call rates for the genotyping of the SNPs were >95%. In order to verify whether there was genotyping error, we randomly selected 40 samples for each SNP, and verified the genotype using Sanger sequencing.

### 2.3. Data Collection

Data were collected by a comprehensive review of hospital records, including fasting plasma glucose (FPG), triglycerides (TG), total cholesterol (TC), low-density lipoprotein cholesterol (LDL-C), systolic blood pressure (SBP), diastolic blood pressure (DBP) and uric acid (UA), which were all tested through standard ways in the clinical laboratory of the Jidong Oil Field Hospital [17]. Weight and height were measured when the participants were lightly clothed and barefoot. Body mass index (BMI) was calculated as weight in kilograms divided by the squared of height in meters (kg/m^2^).

### 2.4. Statistical Analysis

Analyses were conducted with SPSS Software V.18.0 (SPSS Inc., Chicago, IL, USA). Continuous variables were presented as mean ± standard deviation (SD). Categorical variables were presented as numbers and percentages. Student’s *t* test was used to test between-group differences for continuous variables. Chi-square test was applied for categorical variables. Gene-disease associations were measured using odds ratios (ORs), 95% confidence interval (95% CI) and *p* value derived from unconditional logistic regression (ULR) analyses adjusted for age, sex and BMI. Individuals homozygous for the risk allele (R/R), heterozygous (R/r) and homozygous for the non-risk allele (r/r) were assigned a categorical variable according to genotypes. The dominant model was defined as R/R + R/r *versus* r/r, and the recessive model as R/R *versus* R/r + r/r.

The Haploview program 4.2 was used to calculate pairwise linkage disequilibrium (LD) statistics and examine haplotype associations with T2DM. Chi-square test was used to test Hardy-Weinberg equilibrium (HWE) for genotype frequencies. The significance level was set at *p* < 0.05 (two-tailed).

### 2.5. Meta-Analysis

Genetic association studies published before the end of December 2015 were identified through a search of PubMed, EMBASE, ISI Web of Science, Wanfang and Chinese National Knowledge Infrastructure (CNKI). The key words of our search included “*IGF2BP2*”, “T2DM”, “T2D”, rs4402960, rs1470579. Eligible studies had to meet all of the criteria as follows: (1) in case–control or cohort study design; (2) detailed allele frequencies and genotype data for rs4402960, rs1470579 with T2DM risk in Asian populations. The major exclusive criteria:(1) incomplete or duplicate data; (2) meta-analyses and review articles.

According to the meta-analysis following Observation Studies in Epidemiology (MOOSE) standards, data were independently extracted from all genetic association studies independently by two authors [18]. Any disagreement was resolved by discussion with other authors. The items collected were as follows: the first author, the year of publication, cases and controls numbers, country of origin, diagnostic criterion, average BMI and age of cases, frequencies of risk allele and genotypes in cases and controls. The pooled OR and 95% CI were calculated to assess the association between *IGF2BP2* polymorphisms and the risk of T2DM based on the genetic inheritance models described as before. The *Z*-test was used to test the significance of the pooled OR. Subgroup analyses were performed according to the criterion of diagnostic (World Health Organization (WHO) or others), sample size (case samples <500 or >500), and mean BMI (<25 kg/m^2^ or 25–30 kg/m^2^) and age (<55, 55–60 or >60)of cases. Heterogeneity between studies was evaluated with the *I*^2^ test and the Q statistic [19]. *I*^2^ > 50% or *p* < 0.10 for Q statistics was considered as significant heterogeneity. Upon the existence of heterogeneity, either the random-effect model or fixed-effect model would be adopted. Sensitivity analyses were performed for the effect of included individual researches and the assessment of the results stability. Funnel plots represented publication bias graphically [20]. The significance level was set at *p* < 0.05 (two-tailed). Analyses were conducted with the Review Manager 5.0 (Cochrane Collaboration, London, UK).

## 3. Results

### 3.1. Case-Control Study

In total, 274 male and 187 female (53.48 ± 11.33 years) patients, and 249 male and 185 female (51.82 ± 12.67 years) controls were included in the final analysis. T2DM patients had significantly higher BMI, FPG, TG, TC, LDL-C, SBP, DBP than health controls (*p* < 0.05) (Table 1).

In order to verify whether there was genotyping error, PCR and Sanger sequencing were performed for randomly selected 40 samples. The Sanger sequencing data was consisted with MALDI-TOF MS sequencing data (Table S1).

Table 2 listed the genotype and allele frequencies of the rs4402960 and rs1470579 polymorphisms. The genotypic distribution for each of the SNPs was in agreement with the predicted HWE values in health control (rs4402960 HW-*p* = 0.088; rs1470579 HW-*p* = 0.052). The genotype and allele frequencies of the rs4402960 and rs1470579 were similar in T2DM and control groups (*p* > 0.05). In the recessive model, the carriers of TT at rs4402960 had a higher risk of T2DM compared to the carriers of TG + GG (adjusted odds ratio (AOR) = 1.962, 95% CI = 1.065–3.612, *p* = 0.031); the carriers of CC at rs1470579 were more susceptible to T2DM than the carriers of CA + AA (AOR = 2.014, 95% CI = 1.114–3.642, *p* = 0.021). There was no statistical significance found in the dominant model of the rs4402960 and rs1470579 (*p* > 0.05). The two SNPs are in strong LD (D′ = 1 and r^2^ = 0.9). There was no statistical significant association of haplotype with T2DM (Table S2).

### 3.2. Meta-Analysis

**Figure 1:**
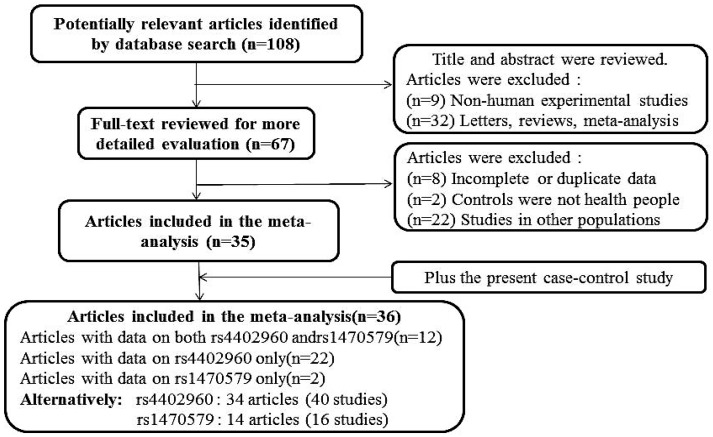
Flow chart of the literature search and study selection.

In total, 36 previous studies plus our case-control study were finally included in this meta-analysis. For the rs4402960, 34 articles were available, including 49,974 cases and 54,315 controls in total. For the rs1470579, 14 articles involved 23,470 cases and 23,671 controls in total [12,15,21,22,23,24,25,26,27,28,29,30,31,32,33,34,35,36,37,38,39,40,41,42,43,44,45,46,47,48,49,50,51,52,53] (Table S3; Figure 1).

The significant associations between rs4402960 polymorphism of *IGF2BP2* and susceptibility of T2DM were found in the allele comparison, genetic models of dominant and recessive(OR = 1.16, 95% CI = 1.13–1.19, *p* = 10^−5^; OR = 1.19, 95% CI = 1.15–1.24, *p* = 10^−5^; OR = 1.24, 95% CI = 1.17–1.32, *p* = 10^−5^, respectively). In the stratified analysis by ethnicity, significantly increased risks were found among Chinese and Japanese populations (*p* < 0.05). However, no significant associations were found in Indian and Korean populations under the recessive model and among other ethnic populations (*p* > 0.05). In the stratified analysis by diagnostic criterion, sample size and average age and BMI of cases, significant associations were identified for rs4402960 in all genetic models (Table 3).

For the association between susceptibility of T2DM and rs1470579 polymorphism, the pooled per-allele OR of the C variant for T2DM was 1.14 (95% CI = 1.11–1.18, *p* = 10^−5^), with corresponding results under dominant and recessive genetic models of 1.11 (95% CI = 1.03–1.19, *p* = 4 × 10^−3^) and 1.21 (95% CI = 1.09–1.36, *p* = 6 × 10^−4^), respectively. In the stratified analysis by ethnicity, significantly increased risks were found among Chinese and Japanese populations (*p* < 0.05). No significant associations were found in the Indian populations (*p* > 0.05). C allele of rs1470579 was also associated with increased T2DM risk, when studies were stratified for diagnostic criterion, size of sample and average BMI of cases (*p* < 0.05). However, under the dominant model, no significant association was found in the “others” of diagnostic criterion, case samples <500 and 25 ≤ BMI ≤ 30 subgroups (*p* > 0.05). Under the recessive model, no significant association was found in the 25 ≤ BMI ≤ 30 subgroup (*p* > 0.05). Upon the research stratification for average age of cases, significant association was not detected in the age <55 subgroup in all genetic models of rs1470579 (*p* > 0.05) (Table 4).

Significant heterogeneity was observed for the rs4402960 polymorphism in allele contrast genetic model and for the rs1470579 polymorphism in dominant and recessive genetic models. Thus, the calculation of the pooled OR was based on a random-effects model. A sensitivity analysis was conducted to explore the sources of heterogeneity, when the number of studies ≥10. There was no single research that affected the pooled OR and CIs in a qualitative way. The funnel plot revealed no obvious publication bias (Figures S1 and S2).

## 4. Discussion

The associations between *IGF2BP2* polymorphism and T2DM risk have been well investigated in several studies, while the association remained unclear. In this study, we validated the associations in a northern Han Chinese population. Our case-control study showed that rs4402960 and rs1470579 polymorphism associated with risk of T2DM under the recessive genetic models (*p* < 0.05), with no statistical significance in other models.

When it comes to the problem of small sample sizes and insufficient statistical power, Meta-analysis was usually performed in terms of genetic association studies of complicated diseases [54]. Therefore, for the further investigation of the influence of the rs4402960 and rs1470579 polymorphisms on T2DM risk, a meta-analysis of 36 publications in the Asians has been conducted with the results of case-control study. As far as we know, this is the first quantitative meta-analysis to date investigating the association between the two SNPs of *IGF2BP2* and T2DM risk in the Asians. The results of meta-analysis revealed that rs4402960 and rs1470579 was significantly associated with susceptibility of T2DM in Asians. Considering small deviation from different genetic models of *IGF2BP2* polymorphism, we investigated the associations of the two SNPs with T2DM risk based on dominant and recessive models and also found a significant result [55]. The significant associations of *IGF2BP2* polymorphism and T2DM were only found under the recessive genetic models, which could be caused by the small affected genotypes and insufficient statistical power in some models. In addition, some studies showed an association of the *IGF2BP2* variant with T2DM under the recessive model [26,40], while others found the under the dominant genetic model [27,34,37,51,56]. Complex diseases such as T2DM are multifactorial and polygenic, and it is generally assumed that each of the factors and genes contribute a small amount to phenotypic variability. In addition, genetic heterogeneity is common for complex traits, referring to the presence of a variety of genetic defects that cause the same disease in clinical settings. Therefore, the polygenic etiology and genetic heterogeneity may partly explained the inconsistent associations between *IGF2BP2* (rs4402960 and rs1470579) and T2DM under various models and different populations.

In the stratified analysis by ethnicity, statistically significant associations were found in Chinese and Japanese for the rs4402960 and rs1470579 polymorphisms in all genetic models. However, we observed that association between rs4402960 polymorphism and risk for T2DM susceptibility in Japanese populations (OR = 1.18) was stronger than that in Chinese (OR = 1.15). Such result may be due to ethnic differences, since the frequency of T allele in rs4402960 is 0.239 in Chinese, while 0.298 in Japanese. Similar results were also found in rs1470579 polymorphism. No significant associations found in other ethnic populations could be explained by study design or the limited number of studies which had insufficient statistical power to detect a slight effect. The more researches are needed to examine the effect of this variation on T2DM in different ethnic populations.

As for the subgroup analysis based on diagnostic criterion, size of sample and average and BMI of cases, significant associations between rs4402960 and T2DM were consisted among all genetic models. The large studies usually dominate the meta-analysis, while the effect of studies with small sample is apparently decreased [57]. In our research, “T” allele in rs4402960 was significantly associated with T2DM risk in both case samples <500 and >500 subgroup. Obesity is an key factor in the onset and progression of insulin resistance and T2DM [58]. It is well known that T2DM and obesity have a genetic basis [59]. Previous studies also have reported significant associations of rs4402960 with obesity [60,61,62]. But we found significant association in both BMI < 25 kg/m^2^ and 25–30 kg/m^2^ studies, suggesting that rs4402960 is an associated factor for T2DM independent of obesity.

For rs1470579, the study showed inconsistent association and significant heterogeneity among the stratified analysis, might resulting from the limited amount of studies available. Older age is risk factor for T2DM [4]. The effect conferred by the risk allele changed with aging [21]. We found rs1470579 significantly associated with T2DM in the mean age >60 studies, while no association existed in the mean age <55. There may be interaction between age and *IGF2BP2*. The association needs to be verified in more studies with larger sample size and prospective study in the future.

*IGF2BP2*, a significant signaling molecule for growth and insulin, has been found have effect on pancreatic development in animal models [63,64]. Huang *et al.* stated that the higher levels of FPG, postprandial serum insulin and TC were founded among T2DM patients who carried the C allele of rs1470579 compared with AA carriers [27]. Rs4402960 attenuate the first phase of glucose-stimulated insulin secretion based on hyperglycaemic clamps [10]. Similar results were also observed in subsequent studies in other populations [25,65,66]. Thus, it could be said that the polymorphism *IGF2BP2* play role in the regulation of pancreatic beta-cell function [27]. Since the location of the two SNPs is the region of 50-kb of intron 2, it can be hypothesized that the effect of diabetes-predisposing variants can be exerted on the expression regulation of *IGF2BP2* [7]. Besides, SNPs could be linked to nearby variants that have a role on larger non-coding transcripts, microRNAs and so on [9]. In addition, rs4402960 and rs1470579 may be a proxy marker rather than a true functional variant. The diacylglycerol kinase g-1 (*DGKG*) located closed to *IGF2BP2*, has been reported associated with metabolic regulation [67]. Therefore, further functional studies of pathophysiological mechanisms *IGF2BP2* are warranted.

Some limitations still existed in the present analysis. Firstly, the study is based on the design of case-control, which restricted the cause-effect inference. The cohort studies are required for the further consequence confirmation. Secondly, only the sample size and allele frequency were available in some of the included studies. Therefore we exhibited the wt/mutant allele numbers in cases and controls in the pooled data, instead of the genotype information. Thirdly, there was still heterogeneity in the subgroup analysis. The more confounding factors should be considering. Fourthly, numerous environmental, genetic factors and the interactions among these factors contribute to the progression of T2DM. Our meta-analysis results did not adjust any confounding covariant, for example gender, drinking status, smoking habit. A more comprehensive analysis should be conducted when more original information is available and interactions among the risk factors are considered.

## 5. Conclusions

In conclusion, we detected the genetic susceptibility of *IGF2BP2* on T2DM under different genetic models, and we confirmed the association between rs4402960 and rs1470579 (*IGF2BP2*) and T2DM under the recessive model in a northern Han Chinese population. Meta-analysis indicated significant contributions of *IGF2BP2* gene rs4402960 and rs1470579 polymorphisms to T2DM in Asian populations.

## Figures and Tables

**Table 1:** Demographic information and clinical characteristics of the participants.

Characteristic	T2DM	Controls	*p*
Gender (male/female)	274/187	249/185	0.53
Age (years)	53.48 ± 11.33	51.82 ± 12.67	0.039
BMI (kg/m^2^)	27.05 ± 3.96	24.65 ± 3.57	<0.001
FPG (mmol/L)	7.26 ± 2.49	6.20 ± 1.95	<0.001
TG (mmol/L)	2.15 ± 1.96	1.63 ± 1.19	<0.001
TC (mmol/L)	4.81 ± 1.13	4.65 ± 0.93	<0.001
LDL-C (mmol/L)	2.79 ± 0.75	2.56 ± 0.64	<0.001
SBP (mmHg)	141.41 ± 20.97	128.33 ± 20.92	<0.001
DBP (mmHg)	87.79 ± 13.40	81.69 ± 13.72	<0.001
UA (mmol/L)	292.95 ± 80.69	300.25 ± 83.93	0.19

BMI: body mass index; FPG: fasting plasma glucose; TG: triglycerides; TC: total cholesterol; LDL-C: low-density lipoprotein cholesterol; SBP: systolic blood pressure; DBP: diastolic blood pressure; UA: uric acid.

**Table 2:** Genotype and allele frequencies of the SNPs in patients with T2DM and healthy controls.

*IGF2BP2* Polymorphisms	Cases	Controls	Crude Model		Adjusted Model *	
			OR (95% CI)	*p*	OR (95% CI)	*p*
**rs4402960**	*n* = 457	*n* = 420				
Allele (%)						
T	230(25.2)	209 (24.9)				
G	914(74.8)	631 (75.1)	1.02 (0.82–1.26)	0.89	1.04 (0.82–1.31)	0.76
Genotype(%)						
GG	261 (57.1)	230 (54.8)				
TG	162 (35.5)	171 (40.7)	0.84(0.63–1.11)	0.20	0.84 (0.62–1.13)	0.24
TT	34 (7.4)	19 (4.5)	1.58(0.88–2.84)	0.13	1.73 (0.92–3.23)	0.09
Dominant model (%)						
GG	261 (57.1)	230 (54.8)				
TT + TG	196 (42.9)	190 (45.2)	0.91(0.70–1.19)	0.48	0.93 (0.70–1.24)	0.63
Recessive model (%)						
TG + GG	423 (93.6)	401 (95.5)				
TT	34 (7.4)	19 (4.5)	1.70 (0.95–3.02)	0.07	1.96 (1.07–3.61)	**0.03**
**rs1470579**	*n* = 459	*n* = 419				
Allele (%)						
A	674 (73.4)	621 (74.1)				
C	244 (26.6)	217 (25.9)	1.04(0.84–1.28)	0.75	1.05 (0.83–1.31)	0.73
Genotype(%)						
AA	253 (55.1)	222 (53)				
CA	168 (36.6)	177 (42.2)	0.82(0.63–1.10)	0.19	0.83 (0.62–1.11)	0.21
CC	38 (8.3)	20 (4.8)	1.67 (0.94–2.95)	0.08	1.76 (0.95–3.23)	0.07
Dominant model (%)						
AA	253 (55.1)	222 (53)				
CC + CA	206 (44.9)	197 (47)	0.92 (0.70–1.20)	0.53	0.93 (0.70–1.23)	0.62
Recessive model (%)						
AA + CA	421 (91.7)	399 (95.2)				
CC	38 (8.3)	20 (4.8)	1.80 (1.03–3.15)	**0.04**	2.01 (1.11–3.64)	**0.02**

* Adjusted for age, sex, BMI.

**Table 3:** Results of meta-analysis for *IGF2BP2* rs4402960 polymorphism and T2DM.

Overall and Subgroup	Number of Studies	T Allele			Number of Studies	Dominant Model			Number of Studies	Recessive Model		
		OR (95% CI)	*p*(Z)	*p*(Q)		OR (95% CI)	*p*(Z)	*p*(Q)		OR (95% CI)	*p*(Z)	*p*(Q)
All	40	1.16 (1.13–1.19)	10^−5^	0.04	22	1.19 (1.15–1.24)	10^−5^	0.59	22	1.24 (1.17–1.32)	10^−5^	0.53
Ethnicity												
Chinese	20	1.15 (1.1–1.2)	10^−5^	0.01	9	1.16 (1.08–1.25)	10^−4^	0.35	9	1.28 (1.16–1.41)	10^−5^	0.4
Japanese	10	1.18 (1.14–1.23)	10^−5^	0.57	9	1.21 (1.15–1.28)	10^−5^	0.93	9	1.24 (1.14–1.35)	10^−5^	0.34
Korean	2	1.15 (1.08–1.23)	10^−5^	0.69	2	1.17 (1.07–1.27)	7 × 10^−4^	0.74	2	1.16 (0.98–1.36)	0.08	0.76
Indian	5	1.17 (1.08–1.27)	10^−5^	0.74	2	1.31 (1.09–1.58)	4 × 10^−3^	0.52	2	1.11 (0.91–1.36)	0.29	0.98
others	3	1.09 (0.99–1.21)	0.09	0.48								
Diagnostic criterion												
WHO	24	1.15 (1.11–1.19)	10^−5^	0.008	15	1.19 (1.15–1.24)	10^−5^	0.75	15	1.25 (1.17–1.33)	10^−5^	0.36
Others	15	1.17 (1.13–1.21)	10^−5^	0.57	7	1.18 (1.05–1.33)	6 × 10^−3^	0.19	7	1.24 (1.07–1.42)	0.003	0.59
Sample size												
<500	9	1.1 (1.02–1.18)	0.01	0.55	5	1.11 (1.01–1.4)	0.04	0.5	5	1.48 (1.14–1.92)	0.003	0.59
>500	30	1.16 (1.13–1.19)	10^−5^	0.01	18	1.2 (1.16–1.25)	10^−5^	0.67	17	1.23 (1.16–1.31)	10^−5^	0.5
Mean BMI of cases												
<25	10	1.21 (1.16–1.27)	10^−5^	0.41	8	1.21 (1.14–1.3)	10^−5^	0.88	8	1.31 (1.17–1.46)	10^−5^	0.44
25–30	22	1.16 (1.13–1.19)	10^−5^	0.69	13	1.18 (1.13–1.24)	10^−5^	0.2	13	1.25 (1.14–1.36)	10^−5^	0.54
Mean age of cases												
<55	10	1.15 (1.1–1.2)	10^−5^	0.51	7	1.18 (1.1–1.28)	10^−4^	0.26	7	1.17 (1.03–1.32)	0.02	0.71
55–60	12	1.2 (1.15–1.24)	10^−5^	0.33	5	1.21 (1.13–1.29)	10^−5^	0.42	5	1.41 (1.24–1.61)	10^−5^	0.43
>60	12	1.16 (1.12–1.2)	10^−5^	0.42	10	1.19 (1.13–1.25)	10^−5^	0.6	10	1.22 (1.12–1.32)	10^−5^	0.6

*p*(Z): Z test used to determine the significance of the overall OR; *p*(Q): Cochran’s chi-square Q statistic test used to assess the heterogeneity in subgroups.

**Table 4:** Results of meta-analysis for *IGF2BP2* rs1470579 polymorphism and T2DM.

Overall and Subgroup	Number of Studies	C Allele			Number of Studies	Dominant Model			Number of Studies	Recessive Model		
		OR (95% CI)	*p*(Z)	*p*(Q)		OR (95% CI)	*p*(Z)	*p*(Q)		OR (95% CI)	*p*(Z)	*p*(Q)
All	16	1.14 (1.11–1.18)	10^−5^	0.65	8	1.11 (1.03–1.19)	0.004	0.09	8	1.21 (1.09–1.36)	6 × 10^−4^	0.05
Ethnicity												
Chinese	9	1.11 (1.06–1.16)	10^−5^	0.63	4	1.04 (0.93–1.16)	0.45	0.16	4	1.14 (0.92–1.4)	0.23	0.25
Japanese	6	1.17 (1.13–1.21)	10^−5^	0.73	3	1.16 (1.04–1.28)	5 × 10^−3^	0.09	3	1.31 (1.12–1.53)	6 × 10^−4^	0.02
Indian	1	1.10 (0.95–1.28)	0.2	NA	1	1.19 (0.93–1.52)	0.16	NA	1	1.1 (0.86–1.4)	0.45	NA
Diagnostic criterion												
WHO	9	1.14 (1.1–1.18)	10^−5^	0.6	4	1.15 (1.06–1.25)	9 × 10^−4^	0.11	4	1.17 (1.03–1.34)	0.02	0.16
Others	7	1.15 (1.09–1.21)	10^−5^	0.43	4	0.99 (0.86–1.15)	0.94	0.36	4	1.3 (1.07–1.58)	0.008	0.05
Sample size												
<500	5	1.17 (1.06–1.29)	0.002	0.25	4	1.01 (0.86–1.17)	0.97	0.1	4	1.57 (1.19–2.08)	0.001	0.22
>500	11	1.14 (1.11–1.17)	10^−5^	0.74	4	1.14 (1.05–1.23)	0.001	0.27	4	1.16 (1.02–1.3)	0.02	0.15
Mean BMI of cases												
<25	6	1.17 (1.13–1.21)	10^−5^	0.73	3	1.16 (1.04–1.28)	0.005	0.09	3	1.31 (1.12–1.53)	6 × 10^−4^	0.02
25–30	9	1.11 (1.06–1.16)	10^−5^	0.62	5	1.07 (0.96–1.18)	0.21	0.19	5	1.12 (0.96–1.31)	0.16	0.39
Mean age of cases												
<55	3	1.11 (0.99–1.24)	0.08	0.34	3	1.14 (0.97–1.33)	0.12	0.08	3	1.19 (0.97–1.47)	0.1	0.28
55–60	4	1.16 (1.09–1.24)	10^−5^	0.54	1	0.88 (0.68–1.15)	0.36	NA	1	1.93 (1.26–2.95)	0.002	NA
>60	8	1.14 (1.11–1.18)	10^−5^	0.39	4	1.13 (1.04–1.23)	0.005	0.23	4	1.16 (1.01–1.34)	0.03	0.09

*p(Z)*: Z test used to determine the significance of the overall OR; p(Q): Cochran’s chi-square Q statistic test used to assess the heterogeneity in subgroups. NA: not available.

## Supplementary Materials

The following are available online at www.mdpi.com/1660-4601/13/6/574/s1, Table S1. Sanger sequencing of randomly selected40 samples. Table S2. Association analysis of *IGF2BP2* haplotypes with T2DM. Table S3. General characteristics of studies evaluating the relationship of *IGF2BP2* rs4402960, rs1470579 polymorphism and T2DM. Figure S1. Funnel plot of the meta-analysis of *IGF2BP2* rs4402960 polymorphism with susceptibility to T2DM (T *vs.* G). Figure S2. Funnel plot of the meta-analysis of IGF2BP2 rs1470579 polymorphism with susceptibility to T2DM (C *vs.* A).
