# Supplementary material for: Association between IGF2BP2 Polymorphisms and Type 2 Diabetes Mellitus: A Case–Control Study and Meta-Analysis
Source: Int J Environ Res Public Health. 2016 Jun 9;13(6):574. doi: 10.3390/ijerph13060574 (PMC4924031; doi:10.3390/ijerph13060574)
Supplement: Supplementary file 1 [file ijerph-13-00574-s001.pdf]

Supplementary Materials: Association between *IGF2BP2* Polymorphisms and Type 2 Diabetes Mellitus: A Case–Control Study and Meta-Analysis

Ping Rao, Hao Wang, Honghong Fang, Qing Gao, Jie Zhang, Manshu Song, Yong Zhou, Youxin Wang and Wei Wang

Table S1. Sanger sequencing of randomly selected40 samples.

	rs4402960		rs1470579	
**No. of Samples**	**Direct Sequencing Data**	**Sanger Sequencing Data**	**Direct Sequencing Data**	**Sanger Sequencing Data**
1	GG	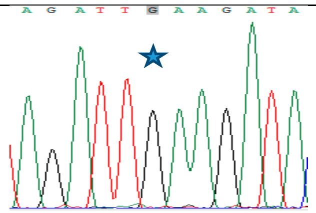	AA	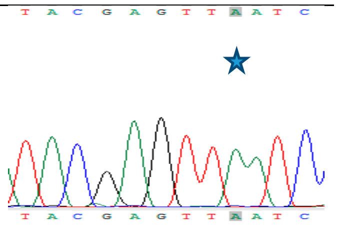
2	GG	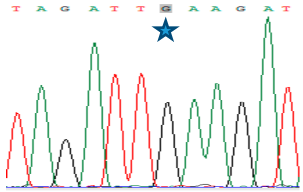	AA	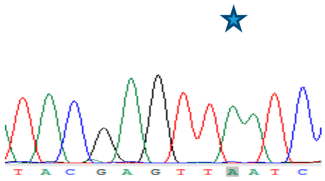
3	GG	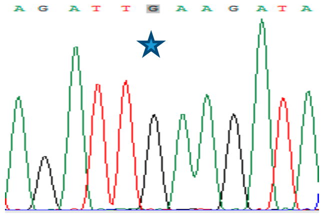	AA	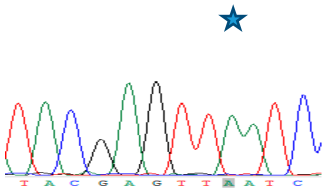
4	GG	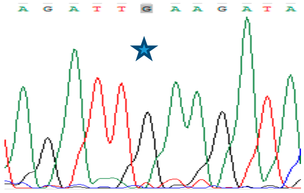	AA	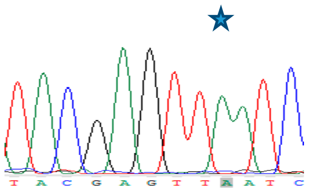
5	GG	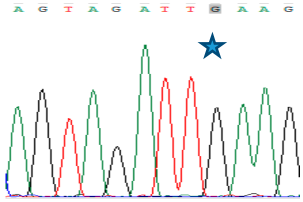	AA	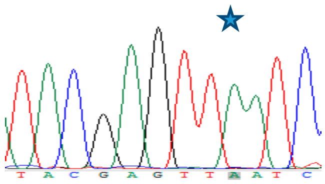
6	GG	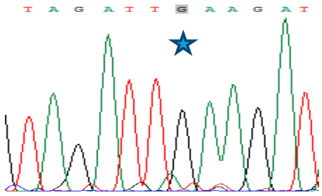	AA	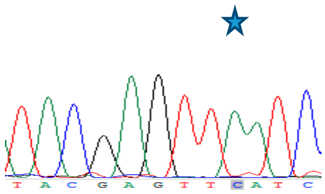
7	GG	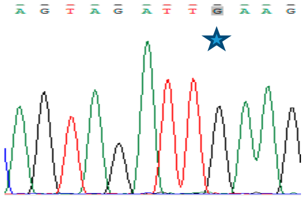	AC	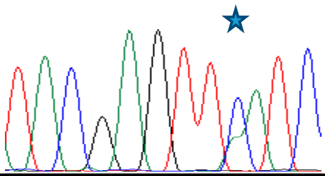

8	GG	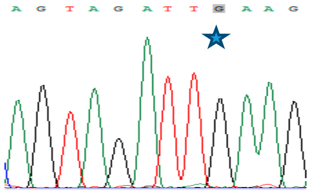	AA	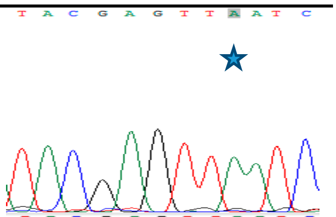
9	GT	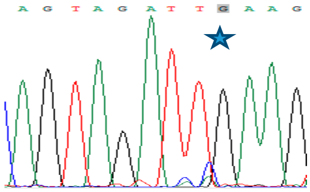	AA	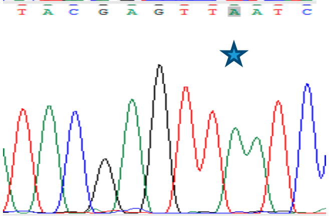
10	GG	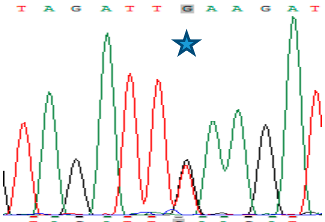	AC	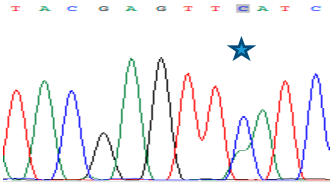
11	GT	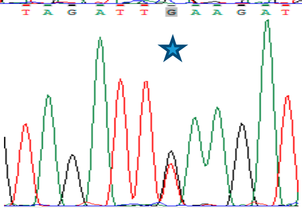	AA	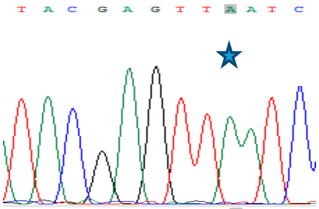
12	GG	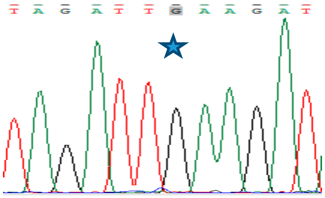	AC	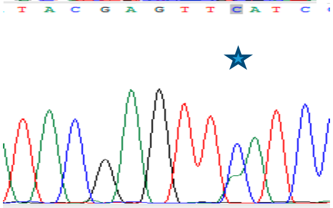
13	GG	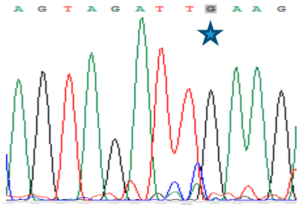	AA	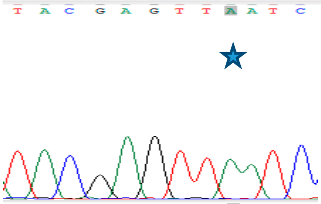
14	GG	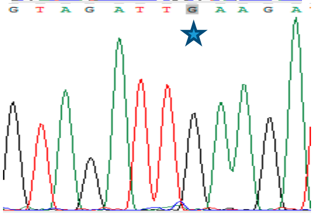	AA	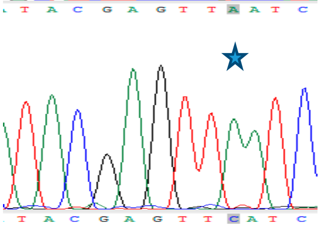
15	GT	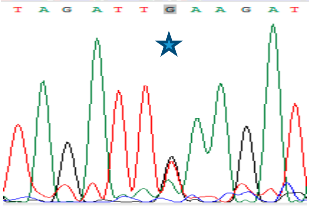	AC	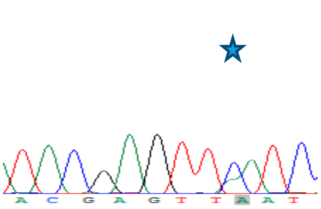
16	GG	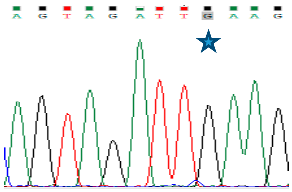	AA	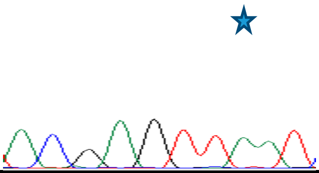

17	GG	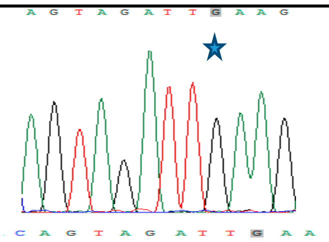	AC	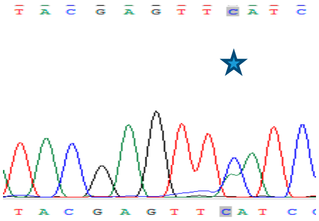
18	GG	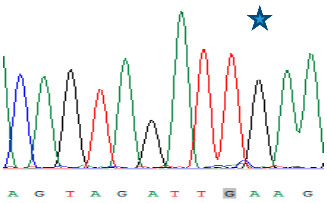	AC	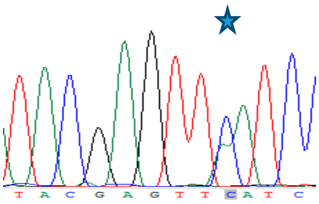
19	GT	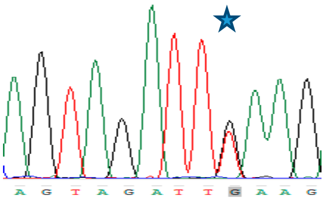	AC	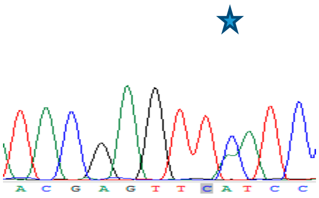
20	GT	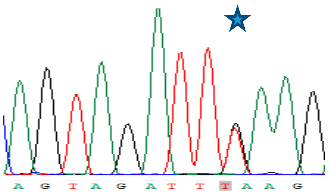	AC	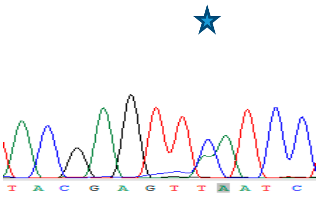
21	TT	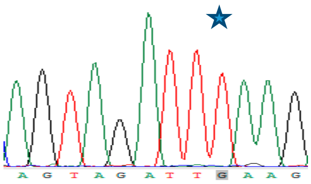	AA	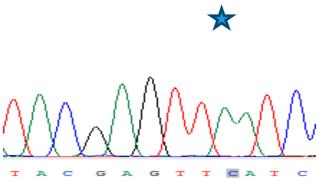
22	GG	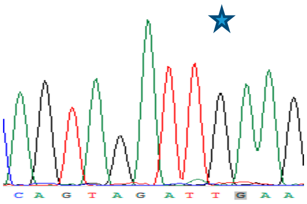	AC	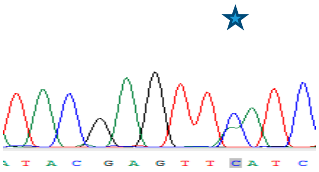
23	GT	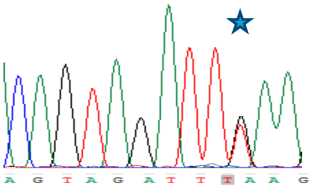	AC	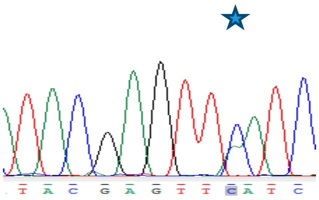
24	TT	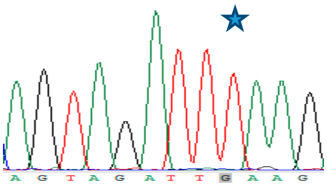	CC	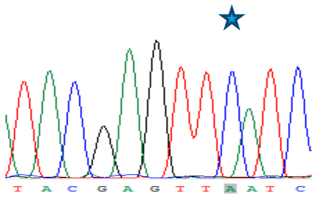
25	GG	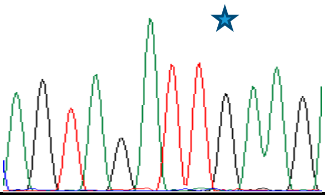	AA	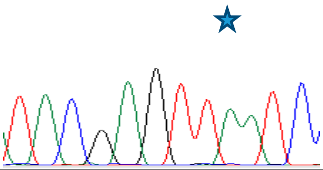

26	GT	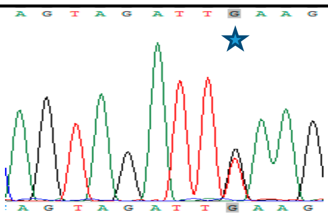	AC	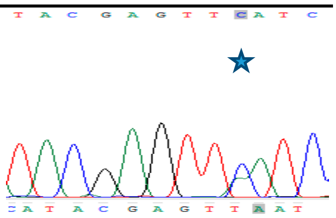
27	GG	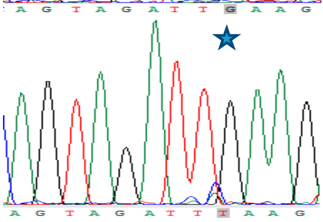	AA	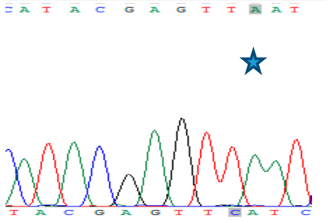
28	GT	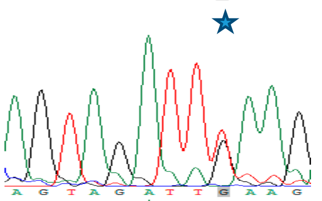	AC	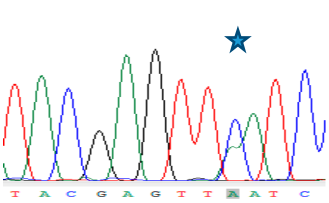
29	GG	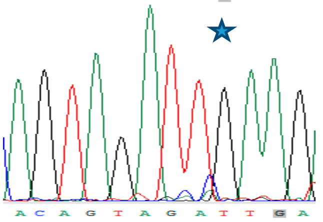	AA	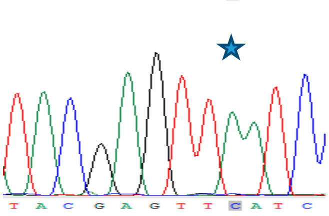
30	GT	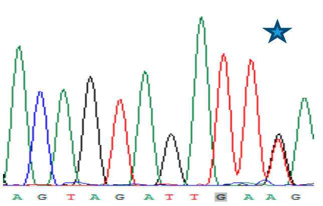	AC	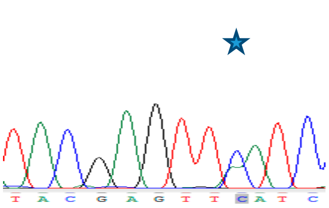
31	GG	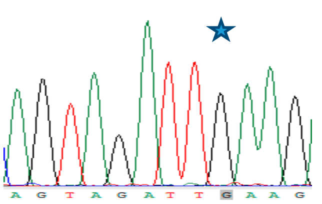	AC	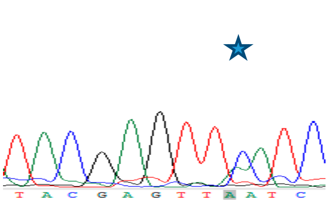
32	GT	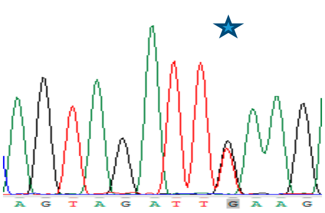	AA	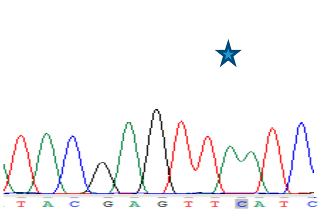
33	GT	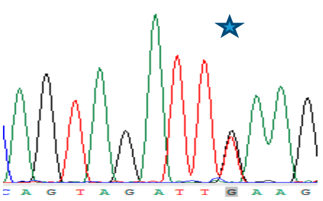	CC	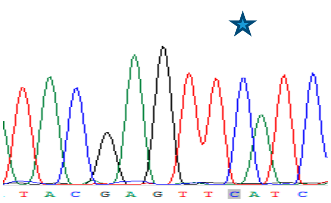
34	GG	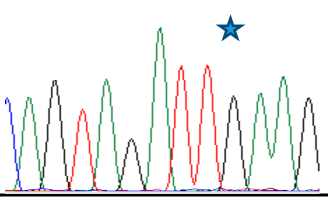	AA	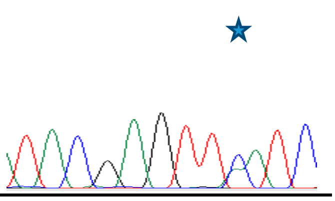

35	GG	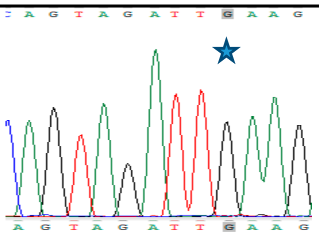	AA	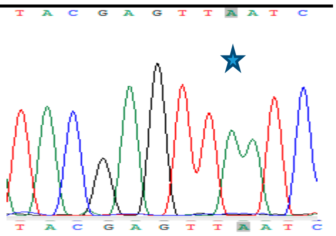
36	GT	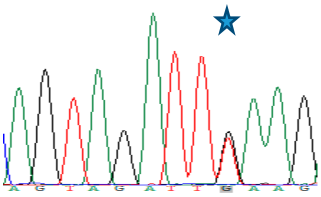	AA	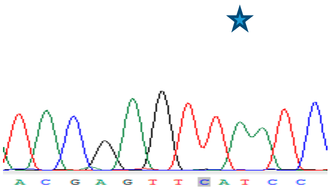
37	GG	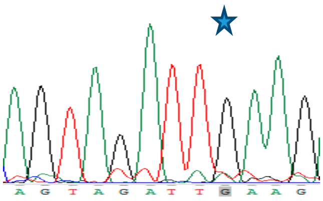	AC	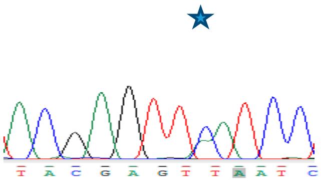
38	GG	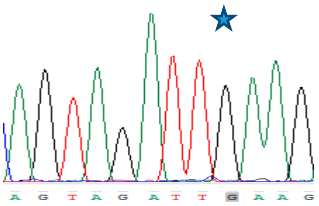	AA	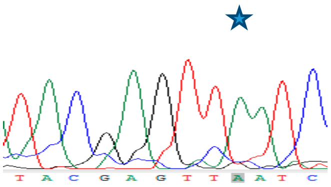
39	GT	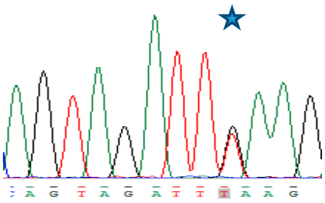	AA	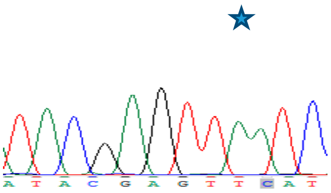
40	TT	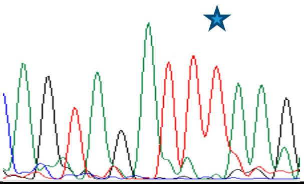	AC	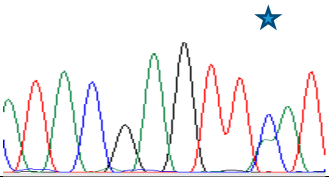

Table S2. Association analysis of *IGF2BP2* haplotypes with T2DM.

SNPs	Haplotype	Control	T2DM	χ^2	p
IGF2BP2	G–A	0.74	0.73	0.14	0.71
(rs4402960-rs1470576)	T–C	0.25	0.25	0.05	0.83
	G–C	0.01	0.01	0.49	0.49

Table S3. General characteristics of studies evaluating the relationship of *IGF2BP2* rs4402960, rs1470579 polymorphism and T2DM.

Author	Year	Ethnicity	Diagnosis	Mean Age, Years		Mean BMI, kg/m^2		rs4402960 (T/G) *				rs1470579(C/A) *			
				Case	Control	Case	Control	Case		Control		Case		Control	
Horikoshi	2007	Japanese	WHO	63	70	24	24	548	1148	520	1162	640	1080	591	1123
Wu	2008	Chinese	WHO	60	58	25	24	224	624	920	2896	231	617	939	2877
Lee	2008	Korean	others	58	55	24	22	553	1153	300	698	NA	NA	NA	NA
Sanghera	2008	Indian	others	54	51	28	27	442	586	332	440	NA	NA	NA	NA
Omori	2008	Japanese	WHO	62	46	24	23	1099	2151	614	1480	NA	NA	NA	NA
Horikawa	2008	Japanese	WHO	62	70	24	23	1296	2408	946	2208	1394	2350	1065	2123
Ng-1	2008	Chinese	WHO	50	25	25	21	755	2207	750	2310	NA	NA	NA	NA
Ng-2	2008	Korean	WHO	58	60	25	24	1012	2108	1202	3094	NA	NA	NA	NA
Liu	2008	Chinese	WHO	64	58	25	25	984	2742	981	2959	1043	2721	904	2418
Takeuchi	2008	Japanese	WHO	65	71	24	23	461	855	303	645	NA	NA	NA	NA
Takeuchi_1	2009	Japanese	WHO	67	65	25	23	366	666	310	682	NA	NA	NA	NA
Takeuchi_2	2009	Japanese	WHO	63	71	23	23	1444	686	1242	742	NA	NA	NA	NA
Takeuchi_3	2009	Japanese	WHO	62	64	NA	23	2684	5282	3340	7436	NA	NA	NA	NA
Tabara	2009	Japanese	others	60	59	24	23	328	658	229	569	352	630	235	561
Miyake	2009	Japanese	others	61	68	24	23	1697	3151	1503	3345	1794	3054	1600	3248
Tan_1	2010	Chinese	WHO	NA	NA	NA	NA	771	2311	1010	3382	NA	NA	NA	NA
Tan_2	2010	Malaysian	WHO	NA	NA	NA	NA	667	1485	1712	3812	NA	NA	NA	NA
Tan_3	2010	Indian	WHO	NA	NA	NA	NA	221	271	320	408	NA	NA	NA	NA
Shu	2010	Chinese	others	52	49	27	23	571	1467	821	2599	NA	NA	NA	NA
Han	2010	Chinese	WHO	56	58	25	25	578	1416	470	1490	NA	NA	NA	NA
Xu_1	2010	Chinese	others	63	59	26	24	938	2712	1020	3380	975	2675	1069	3331
Xu_2	2010	Chinese	others	62	61	26	25	28	106	298	1010	29	105	312	1000
Lin	2010	Chinese	WHO	60	58	24	24	184	2874	115	2763	NA	NA	NA	NA
Chauhan_1	2010	Indian	WHO	53	50	27	25	937	1101	845	1167	NA	NA	NA	NA
Chauhan_2	2010	Indian	WHO	46	33	27	20	1467	1467	1538	1806	NA	NA	NA	NA
Wen	2010	Chinese	WHO	60	59	25	24	626	1704	576	1696	NA	NA	NA	NA
Huang	2010	Chinese	WHO	49	48	25	24	190	510	88	326	212	488	102	312
Yamauchi_1	2010	Japanese	WHO	66	53	24	23	NA	NA	NA	NA	3560	6196	2208	4482
Yamauchi_2	2010	Japanese	WHO	64	64	24	24	NA	NA	NA	NA	2118	3654	2087	4087
Rees	2011	Pakistani	WHO	56	56	27	26	1287	1853	1191	1943	NA	NA	NA	NA
Cui	2011	Chinese	WHO	NA	NA	NA	NA	409	1447	392	1220	436	1150	413	1199
Iwata	2012	Japanese	others	65	70	24	24	492	956	443	1083	NA	NA	NA	NA
Li	2012	Chinese	others	58	53	24	24	138	194	41	347	NA	NA	NA	NA

Jia	2012	Chinese	WHO	51	44	25	24	220	524	196	524	NA	NA	NA	NA
Zhang	2013	Chinese	WHO	56	51	26	23	2301	6237	1651	5457	NA	NA	NA	NA
Kommoju	2013	Indian	others	52	50	27	25	773	743	609	633	773	743	603	627
Chen	2013	Chinese	others	NA	NA	NA	NA	416	470	1032	1206	NA	NA	NA	NA
Jiao	2013	Chinese	others	58	NA	NA	NA	504	1322	237	847	NA	NA	NA	NA
Kuo	2013	Chinese	others	65	65	25	25	2404	6666	2294	7306	NA	NA	NA	NA
Li	2013	Chinese	WHO	59	54	25	24	NA	NA	NA	NA	1119	2879	1000	2952
Chang	2014	Chinese	others	60	56	25	24	766	2238	688	2348	784	2220	724	2312
Song	2015	Chinese	WHO	54	56	25	23	27	75	31	71	NA	NA	NA	NA
Al Sinani	2015	Omanis	others	56	43	30	29	794	1190	210	378	NA	NA	NA	NA
This study	2015	Chinese	others	53	52	27	25	230	684	209	631	244	674	217	621

WHO: World Health Organization; BMI: body mass index; NA, not available; * Data are expressed as allele counts of mutant/wild for each examined variant.

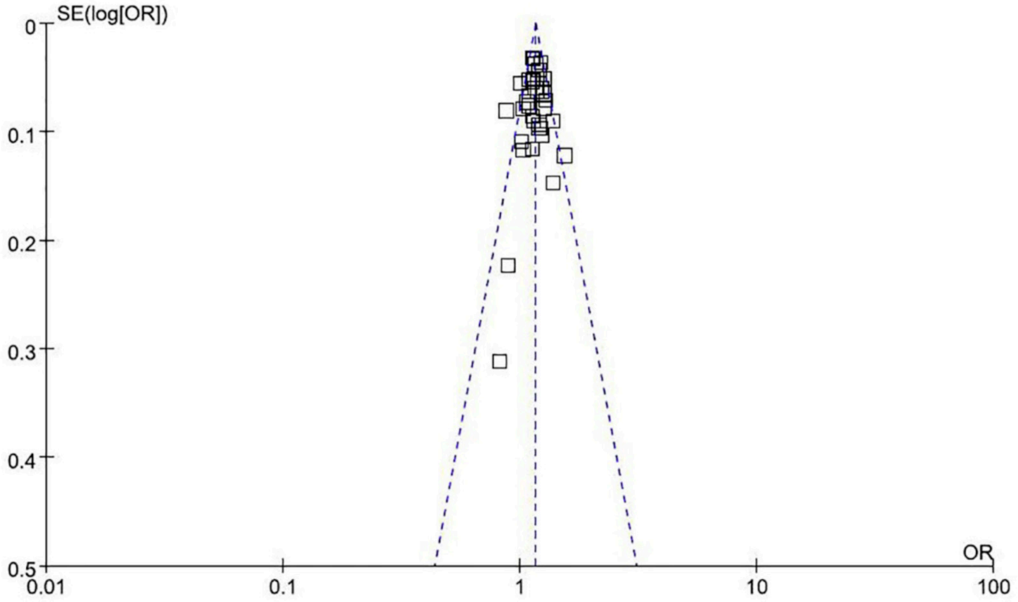

Figure S1. Funnel plot of the meta-analysis of *IGF2BP2* rs4402960 polymorphism with susceptibility to T2DM (T *vs.* G).

Each point represents a separate study for the indicated association. Log[OR], natural logarithm of OR. Horizontal line, mean magnitude of the effect.

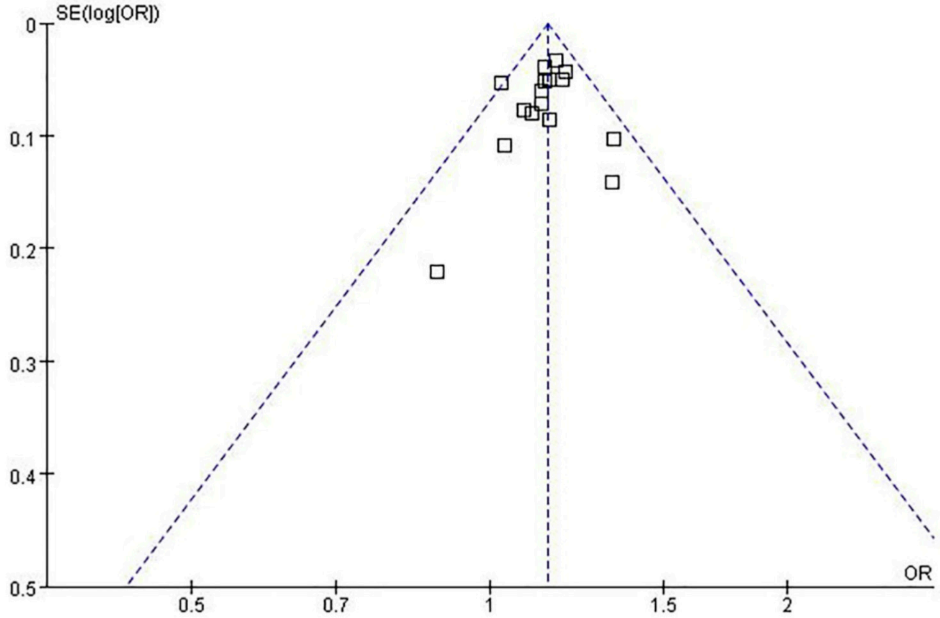

Figure S2. Funnel plot of the meta-analysis of IGF2BP2 rs1470579 polymorphism with susceptibility to T2DM (C *vs.* A).

Each point represents a separate study for the indicated association. Log[OR], natural logarithm of OR. Horizontal line, mean magnitude of the effect.

© 2016 by the authors; licensee MDPI, Basel, Switzerland. This article is an open access article distributed under the terms and conditions of the Creative Commons by Attribution (CC-BY) license (http://creativecommons.org/licenses/by/4.0/).
